# Music, memory and mechanisms in Alzheimer’s disease

**DOI:** 10.1093/brain/awv148

**Published:** 2015-07-16

**Authors:** Camilla N. Clark, Jason D. Warren

**Affiliations:** Dementia Research Centre, UCL Institute of Neurology, University College London, London, UK

## Abstract

**This scientific commentary refers to ‘Why musical memory can be preserved in advanced Alzheimer’s disease’, by Jacobsen *et al.* (doi:10.1093/brain/awv135).**

**This scientific commentary refers to ‘Why musical memory can be preserved in advanced Alzheimer’s disease’, by Jacobsen *et al.* (doi:10.1093/brain/awv135).**

The power of music to unlock memories and other cognitive capacities in Alzheimer’s disease is a cherished tenet of clinical neurology, and music is unquestionably a welcome source of comfort to many people with this devastating illness. Scientific validation of clinical instinct, however, is rarely straightforward. Though ubiquitous, music is hard to study scientifically; until comparatively recently, we lacked the conceptual framework to analyse it as a neuropsychological phenomenon and the practical tools to examine brain structures and processes that allow us to experience it. Thanks in part to the current ascendancy of functional neuroimaging, these tools are now to hand and the time is ripe to take music neuroscience to the clinic. The work of Jacobsen and colleagues reported in this issue of *Brain* (Jacobsen *et al.*, 2015) contributes an interesting fresh perspective on why musical memories might survive the predations of dementia.

To tackle this question, Jacobsen and colleagues first sought to identify areas activated during a musical memory task in healthy young adult brains, using high-field functional MRI and multivariate classification of regional activation patterns. These same brain areas were then assessed in a cohort of patients with Alzheimer’s disease versus a healthy older reference group, using a region of interest approach combined with standard structural (MRI), metabolic (fluorodeoxyglucose-PET) and amyloid ligand (florbetapir-PET) neuroimaging. Certain cortical areas were linked to musical memory in young adults—in particular, the anterior cingulate and the ventral presupplementary motor area, embedded in a more widespread network also including anterior temporal, frontal polar and insular cortices. The key areas implicated in musical memory in the young adults were found to be relatively less affected by Alzheimer’s disease than other areas of cortex, as indexed using standard neuroimaging biomarkers. What can these findings tell us about brain mechanisms of musical memory in Alzheimer’s disease?

The answer may depend, firstly, on the nature of musical memories. The existence of multiple human memory systems mediating the autobiographical record (episodic memory; the context in which a piece was heard), knowledge about the world (semantic memory; recognition of a familiar tune) and learned motor skill sequences (procedural memory; playing an instrument) is well established for a range of sensory phenomena. In a neuropsychological sense, the first two memory systems are ‘explicit’, the last ‘implicit’ and all are forms of ‘long-term’ memory (contrasted with the ‘short term’ rehearsal of information in working memory). For musical entities, limited previous work suggests that these memory systems are anatomically as well as cognitively at least partly dissociable in the normal brain (Platel *et al.*, 2003; see Fig. 1). In the paradigm of Jacobsen and colleagues, the conditions of ‘long known’ and ‘recently known’ songs correspond broadly to musical semantic and episodic memory, respectively; and the most robust data were correlates of ‘long-known’ songs (musical semantic memories, following the formulation above). The functional MRI scanning task was based on a familiarity judgement. Familiarity, while widely adopted as a convenient procedure in music psychology, may not fully capture the rich structure and associations of musical memories (Omar *et al.*, 2010). However, if musical memories are indeed special, this is most likely attributable to their emotional resonance. Our favourite songs transport us largely by conjuring surrogate emotions: the neural apparatus of emotion, reward, autonomic and motor programmes is hard-wired into our experience of music and this may have been the very point of music, in evolutionary terms (Zatorre and Salimpoor, 2013; Clark *et al.*, 2015). Affective salience and motor preparation responses might well be reflected in the activation of anterior cingulate and premotor areas observed by Jacobsen and colleagues.

**Figure 1:**
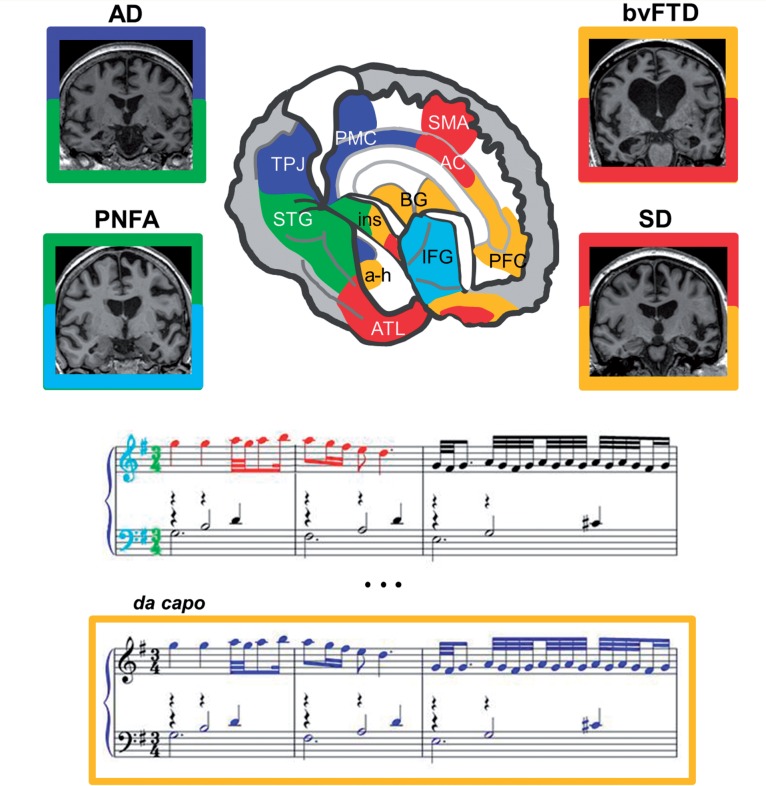
**A framework for analysing the effects of dementias on music cognition.** The cut-away brain schematic (*top centre*) shows cerebral networks associated with components of music cognition, based on clinical and normal functional neuroanatomical evidence, including the work of Jacobsen and colleagues. The right cerebral hemisphere is projected forward in the schematic, however musical functions are bi-hemispherically distributed. Coronal MRI brain sections (*side panels*; right hemisphere shown on the left in each section) represent canonical neurodegenerative syndromes predicted to affect these music networks, with characteristic profiles of regional cerebral atrophy: Alzheimer’s disease (AD), bilateral symmetrical mesial temporal lobe atrophy; behavioural variant frontotemporal dementia (bvFTD), asymmetric (predominantly right-sided) frontal and temporal lobe atrophy; progressive non-fluent aphasia (PNFA), asymmetric (predominantly left-sided) peri-sylvian atrophy; and semantic dementia (SD), asymmetric (predominantly left-sided) anterior temporal lobe atrophy. The musical score (*below*) shows excerpts from the Aria that opens and closes (*da capo*) the ‘Goldberg Variations’ by J.S. Bach. Components relevant to music cognition are colour coded throughout as follows: blue, tracking of musical events and musical episodic memory; green, elementary musical property (e.g. tempo) processing; cyan, scale and key processing; red, recognition of familiar musical motifs (musical semantic memory); gold, musical emotion. The figure illustrates the close cognitive and neuroanatomical relations between musical memory and emotion; the return of the Aria’s simple theme after a long series of 30 increasingly elaborate variations triggers both recall of a musical episode and an emotional highpoint of the Goldbergs for many listeners. AC = anterior cingulate; a-h = amygdala–hippocampus; ATL = anterior temporal lobe; BG = basal ganglia; IFG = inferior frontal gyrus/frontal operculum; ins = insula; PFC = prefrontal cortex; PMC = posterior medial cortex (posterior cingulate, precuneus); SMA = supplementary motor area; STG = superior temporal gyrus; TPJ = temporo-parietal junction.

While it is not entirely clear how musical memories fit within standard formulations of human memory, it is well known that these memory systems are differentially vulnerable to Alzheimer pathology. In general, Alzheimer’s disease initially erodes episodic memory with more variable impairment of semantic memory and relative preservation of procedural memory, at least early on. Whether sparing of musical memory in Alzheimer’s disease should be viewed as paradoxical might therefore depend on which type of memory we mean. The available literature does not resolve this. As Jacobsen and colleagues acknowledge, studies of musical memory in Alzheimer’s disease have been based on small numbers of cases using variable methodology and assessing different kinds of memory, and the extent of any sparing seems rather variable (Omar *et al.*, 2012). Indeed, fine-grained analysis of musical semantic knowledge may expose deficits in Alzheimer’s disease (Omar *et al.*, 2010). On the other hand, non-Alzheimer dementias may constitute an arguably more informative disease model for revealing the fractionation of music cognition (Fig. 1). In the frontotemporal lobar degeneration spectrum, selective disintegration of neuroanatomical and cognitive systems illustrates the involvement of temporal lobe structures in musical semantic memory, but also how it can sometimes be strikingly preserved despite pervasive semantic failure (Omar *et al.*, 2010, 2012; Golden *et al.*, 2015*a*), as well as the behavioural consequences of excessive responsiveness to music (music craving or ‘musicophilia’: Agustus *et al.*, 2015). It would be intriguing if the extratemporal substrates of musical memory identified by Jacobsen and colleagues turned out to be a signature of spared musical memory in semantic dementia as well as (or instead of) Alzheimer’s disease. At the same time, the propensity of music to move us may make it a uniquely potent probe of the so-called ‘default mode network’: this brain system governs the interface of our inner life with the wider world and it is preferentially targeted by Alzheimer’s pathology (Golden *et al.*, 2015*b*).

Jacobsen and colleagues use structure-function relations in the healthy brain to delineate relevant neural circuitry and then use this information to draw inferences about the effects of disease. A similar logic led to the recognition that pathogenic proteins can spread over large-scale intrinsic brain networks, an insight that has transformed our picture of neurodegenerative diseases (Warren *et al.*, 2013). However, the logical loop that Jacobsen and colleagues have opened with their provocative title will only be closed by studying patients directly. Functional neuroimaging of the working brain is feasible in patients with Alzheimer’s disease and other dementias, as has recently been shown using musical and other complex auditory stimuli (Agustus *et al.*, 2015; Golden *et al.*, 2015*b*). Clinical neurologists are sometimes sceptical about its value but as a technique for understanding dementia diseases, functional MRI has three main advantages over structural MRI techniques: it can detect disease-associated functional alterations prior to the onset of irrecoverable brain damage, it can measure functional connections between brain regions, and it can uncover aberrant and compensatory increases in brain activity. These advantages mean that functional MRI is well suited for studying the mechanisms by which brain networks break down in dementia and for testing specific pathophysiological hypotheses. There is no doubt, however, that the application of task-related functional MRI is challenging in cognitively impaired patients and is likely to entail customised protocols based on short scanning sessions and minimal in-scanner task demands (Agustus *et al.*, 2015; Golden *et al.*, 2015*b*).

Dementia is much more than the failure of memory. Alzheimer’s and other neurodegenerative diseases have often profound consequences for complex behaviours that impact on the emotional and social functioning of patients in their daily lives. Such phenomena are notoriously difficult to capture using the conventional pencil-and-paper armamentarium of psychometric tests. The field of neurodegeneration research cries out for comprehensive pathophysiological models that will allow disease effects to be understood and anticipated at the level of the whole brain. Music could potentially provide such a model (Fig. 1). As a psychological construct, it is universal but grounded in the quotidian. As a neurobiological phenomenon, it is multidimensional: these dimensions range from the decoding of abstract sensory signals potentially lasting several hours, to physiological responses that shift from moment to moment with sometimes surprising results (chills, tears, the tapping of feet). Furthermore, the components of music lend themselves readily to analysis, and music neuroscience has linked these to distributed brain mechanisms that are also targeted by canonical dementia diseases (Fig. 1). As an essentially network-based brain function, music is likely *a priori* to map the functional derangements and compensations induced by these network-based proteinopathies. Going further, the brain mechanisms that support musical information processing may eventually illuminate the specific neural architectures that underpin particular proteinopathies (Warren *et al.*, 2013).

Few neurologists would dispute that fresh takes on Alzheimer’s disease (and dementia more generally) are urgently needed as the pandemic looms and treatments remain elusive. The work of Jacobsen and colleagues reminds us that seemingly exotic beasts such as musical memory may yet prove to be the black swans that force us to re-evaluate how these diseases work, inspiring bold new hypotheses that will be tested using powerful new methods. The conundrum of Alzheimer’s disease may finally be solved only once we understand its more subtle and least tractable effects, which are frequently the effects that matter most to our patients. Music may be a means to achieving this end.

## Funding

The authors are supported by the Wellcome Trust (Grant No 091673/Z/10/Z).

## References

[awv148-B1] Agustus JL Mahoney CJ Downey LE Omar R Cohen M White MJ Functional MRI of music emotion processing in frontotemporal dementia. Ann N Y Acad Sci 2015; 1337: 232–40. 25773639 10.1111/nyas.12620 PMC4402026

[awv148-B2] Clark CN Downey LE Warren JD Brain disorders and the biological role of music. Soc Cogn Affect Neurosci 2015; 10: 444–52. 24847111 10.1093/scan/nsu079 PMC4350491

[awv148-B3] Golden HL Downey LE Fletcher PD Mahoney CJ Schott JM Mummery CJ Identification of environmental sounds and melodies in syndromes of anterior temporal lobe degeneration. J Neurol Sci 2015a: 352: 94–8. 25843288 10.1016/j.jns.2015.03.007 PMC4425361

[awv148-B4] Golden HL Agustus JL Goll JC Downey LE Mummery CJ Schott JM Functional neuroanatomy of auditory scene analysis in Alzheimer's disease. Neuorimage Clin 2015b; 7: 699–708. 10.1016/j.nicl.2015.02.019 PMC4446369 26029629

[awv148-B5] Jacobsen JH Stelzer J Fritz T Chetelat G LaJoie R Turner R Why musical memory can be preserved in advanced Alzheimer’s disease. Brain 2015. 10.1093/brain/awv135 26041611

[awv148-B6] Omar R Hailstone JC Warren JD Semantic memory for music in dementia. Music Percept 2012; 29: 467–77.

[awv148-B7] Omar R Hailstone JC Warren JE Crutch SJ Warren JD The cognitive organization of music knowledge: a clinical analysis. Brain 2010; 133: 1200–13. 20142334 10.1093/brain/awp345 PMC2850578

[awv148-B8] Platel H Baron JC Desgranges B Bernard F Eustache F Semantic and episodic memory of music are subserved by distinct neural networks. Neuroimage 2003; 20: 244–56. 14527585 10.1016/s1053-8119(03)00287-8

[awv148-B9] Warren JD Rohrer JD Schott JM Fox NC Hardy J Rossor MN Molecular nexopathies: a new paradigm of neurodegenerative disease. Trends Neurosci 2013; 36: 561–9. 23876425 10.1016/j.tins.2013.06.007 PMC3794159

[awv148-B10] Zatorre RJ Salimpoor VN From perception to pleasure: music and its neural substrates. Proc Natl Acad Sci USA 2013; 110 (Suppl 2): 10430–7. 23754373 10.1073/pnas.1301228110 PMC3690607

